# Immobilization of Purified Pectin Lyase from *Pseudomonas putida* onto Magnetic Lily Flowers (*Lilium candidum* L.) Nanoparticles and Applicability in Industrial Processes

**DOI:** 10.3390/molecules25112671

**Published:** 2020-06-09

**Authors:** Esen Tasgin, Hayrunnisa Nadaroglu, Aynur Babagil, Nazan Demir

**Affiliations:** 1 Department of Nutrition and Dietetics, Faculty of Health Sciences, Atatürk University, 25240 Erzurum, Turkey; 2 Department of Nano-Science and Nano-Engineering, Institute of Science and Technology, Ataturk University, 25240 Erzurum, Turkey; ababagil@hotmail.com; 3 Department of Food Technology, Vocational Collage of Technical Science, Ataturk University, 25240 Erzurum, Turkey; 4 Faculty of Science Department of Chemistry, Mugla Sıtkı Koçman University, 48000 Mugla, Turkey; nazdemir@mu.edu.tr

**Keywords:** pectin lyase, triple phase precipitation (TPP), lily (*Lilium candidum* L.), immobilization

## Abstract

Pectinases are an important class of enzymes distributed in many higher plants and microorganisms. One of these enzymes is pectin lyase which has an important role in industrial applications such as clarification of fruit juices. Pectin lyase was purified with 73% yield from *Pseudomonas putida* bacteria and was 220.7-fold using three phase precipitation technique. Molecular weight of purified pectin lyase was determined as 32.88 kDa with SDS-polyacrylamide gel electrophoresis. The pectin lyase was immobilized covalently via the L-glutaraldehyde spacer to the cellulosic structures of lily flowers (*Lilium candidum* L.). The immobilized enzyme was then magnetized by modifying with γ-Fe_3_O_4_ nanoparticles and determined the most appropriate immobilization conditions as pH 6 and 30 °C. Purified pectin lyase was connected to magnetized support material after 60 min at the rate of 86.4%. The optimum pH and temperatures for the free and immobilized pectin lyase was found to be 6.0 and 40 °C. pH and thermal stabilities of the free and immobilized pectin lyase enzyme have been preserved at high-low temperatures and pH. The structural characterization of the immobilized pectin lyase was performed by SEM, FT-IR, and XRD chromatographic analyses and it was observed that the support materials structure was appropriated to immobilization with pectin lyase and to modify with Fe_3_O_4_ nanoparticles.

## 1. Introduction

Recently, interest in biopolymers such as cellulose, starch, lignin, and pectin has been increasing. Pectinases are a large group of enzymes that break down pectin polysaccharides in plant tissues through depolymerization and deesterification reactions and convert them into simpler molecules such as galacturonic acids. Pectinases are produced by a large number of organisms such as bacteria [1,2], fungi [3], and yeasts [4]. It has been reported that most of the pectin lyases studied so far have been obtained from microorganisms and have insufficient presence in plants and animals [5]. Pectinolytic enzymes obtained from microorganisms are of great interest because of their biotechnological potential and their role in industrial applications [6]. One of the most important enzymes in this group, pectin lyase (PL, EC 4.2.2.3) is used for the production of juice and wine, especially in the food industry, owing to its important role in the expansion of the cell wall and in the softening of some plant tissues [7,8]. Processed juices and wines are cloudy due to the suspension of polysaccharide particles such as pectin, cellulose, hemicellulose, starch, and lignin, which are components of plant cell walls [9]. Pectinases reduces blurred view disintegrating its pectic structures and increase the fruit juice yields [1,10]. Pectin lyases (PLs) are obtained by different enzyme separation and purification methods from fungi, bacteria, and yeasts, and because of their high separation properties. The conventional chromatography techniques such as CM-Cellulose and Sephadex G-100 column chromatographies, DEAE-Sepharose ion exchange, have been efficiently used to purify these enzymes [7]. Since these methods are usually multi-stage and cause depolymerization, the triple phase precipitation technique (TPP) is also often preferred in recent years [5,11,12]. The stability of these enzymes depends on the medium, otherwise the enzymes cannot function appropriately and can be easily disrupted. Recent studies have shown that immobilization techniques help maintaining the stability of these enzymes [12,13]. However, the development of new methods and using different supports materials for immobilizing enzymes is important in enzyme technology. The support materials protect the structure of the enzymes against difficult reaction conditions ensuring their activity remains high [14,15]. For this reason, a wide variety of materials of different origin, such as organic, inorganic, and hybrid or composite are being used for enzyme immobilization [16]. Although free pectinases have excellent catalytic properties, immobilization has been carried out in order to maintain their activities for a longer time and to use them over and over again [15]. Biopolymers, which are polymers of natural origin, have been used as alternative support materials for enzymes in recent years. Materials such as chitin-chitosan [17,18], alginate [13,19], collagen, cellulose, agarose [20,21,22] mostly have been preferred as immobilization support biopolymers. Biopolymers possess a unique set of properties such as biodegradability to harmless products, biocompatibility and non-toxicity, high affinity to proteins, which make them suitable supports for enzymes [23]. The main properties of the support materials used for enzyme immobilization are regeneration, reusability, availability, relatively low price, chemical and thermal stability, high affinity for enzymes, and biocompatibility [16]. In addition to carrying these properties of the support materials, bio polymers have also unique properties such as biodegradability, biocompatibility, non-toxic, and high affinity for proteins, making them suitable supports for enzymes [23]. They consist mainly of the large biopolymers cellulose, hemicellulose, lignin, and pectin. Owing to their reactive functional groups such as hydroxyl, amine, and carbonyl due to their natural structure, their interaction with the enzyme is easy and fast [24]. Since these materials are renewable and easy to obtain, they are cheap and their costs associated with the immobilization process are low [25].

In this study, easily accessible and non-toxic lily flower was used for the first time as a support material for immobilization. Although there is no study that the cellulosic structure of the lily flower is a good support for enzyme immobilization, it has been thought that the cellulosic polysaccharide structure of the lily flower may be a potential candidate for immobilization of the pectin lyase enzyme [17]. In previous studies, lily flower has been shown to have antioxidant, anticancer, antifungal, and antivirus properties [26]. The aim of this study is to nano-magnetically alter the lily plant as a new natural support material and to immobilize the pectin lyase enzyme, to characterize it, to investigate reusability conditions, and to determine its effectiveness in clarifying some juice.

## 2. Results and Discussion

### 2.1. Purification of Pectin Lyase from Pseudomonas putida

The pectin lyase enzyme was produced extracellularly from *Pseudomonas putida* bacteria. Purification of the pectin lyase enzyme was performed by a three-phase partitioning technique [11]. First, optimized with n-butanol, the maximum PL enzyme activity was determined according to the activity measurements at a ratio of 1:0.5 (extract:n-butanol). At the optimum concentration of n-butanol, the precipitation of 1. ammonium sulphate was performed between 20–80%. The highest activity was obtained at 60%. The second ammonium sulfate precipitation was performed at 65–75% concentration and the highest activity was determined as 65%. PL enzyme was purified by a three-phase partitioning technique [11], obtaining a 73% yield and 220.7-fold from *Pseudomonas putida* (Table 1).

With the triple phase precipitation technique (TPP) used in purification of pectin lyase, it has been shown that higher yields are obtained compared to the traditional multi-stage separation methods [11]. The high efficiency we obtained with the use of this method has shown that the method used in the purification process of the enzyme is a useful approach.

Three-phase partitioning was used to purify pectinases from *Aspergillus niger* and tomato and the yields of 76% (*Aspergillus niger*) and 183% (tomato) were obtained [27]. The alkaline pectin lyase from *Bacillus cereus* was purified about 24-fold using DEAE and Sephadex G-75 gel filtration chromatography by Kohli and Gupta [13]. In some studies, PL enzyme have been purified also 36.36, 58.01 and 40.8 times from some bacteria such as *Bacillus pumilus* [2], *Aspergillus flavus* [27], *Geobacillus stearothermophilus* [1].

### 2.2. Characterization of Purified Pectin Lyase from Pseudomonas putida

The maximum reaction rate (Vmax), Michaelis constant (K_M_), and catalytic efficiency were determined by measuring initial reaction rates and varying the amount of substrate. The immobilized enzymes showed higher Vmax and lower K_M_ values than the free enzyme (for immobilized enzymes, K_M_, 0.86 mg/mL and Vmax, 23.20 µmol/Lmin). The enzyme kinetic parameters were determined using different substrates and it was observed that pectin is the best substrate. Similarly, in the study of the Babagil, pectin was determined as the most suitable substrate [28]. In addition, the affinities of enzymes immobilized PL (IM-PL) and free PL (F-PL) enzyme were compared against all substrates (the pectin, locust bean gum, and chitin) and it was determined that IM-PL showed higher affinity (Table 2).

### 2.3. Effect of Incubation Time, pH and Temperature to Immobilization of PL Enzyme on MLf

**Figure 1:**
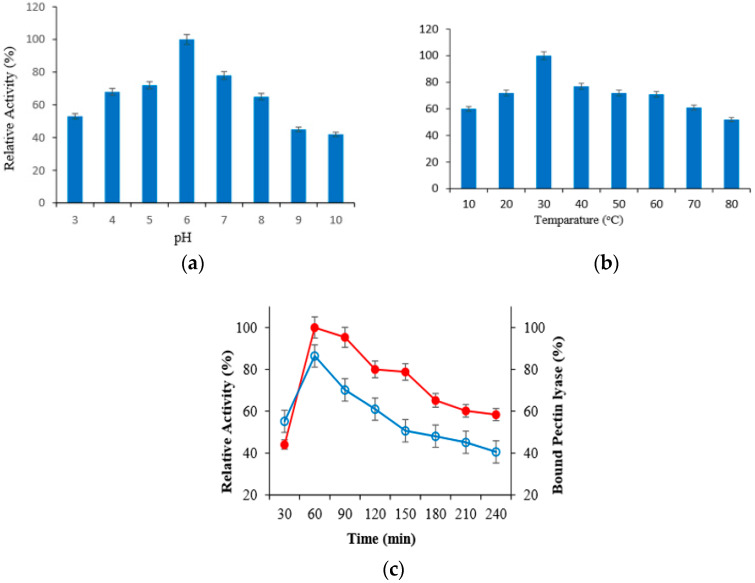
Results of the effect of pH (**a**), temperature (**b**) and incubation time (**c**) on immobilization of PL enzyme on N-MLf.

Optimal pH and temperature was primarily determined to indicate the optimum conditions of covalent immobilization to the surface of magnetized lily flower with nano-Fe_3_O_4_ of purified PL enzyme. Purified PL enzyme was immobilized using appropriate buffers among pH 3–8, and the relative activity (%) of immobilized PL enzyme was calculated. Accordingly, it was determined that the purified PL enzyme was connected to support material at the high level at pH 6 and 30 °C. Then, the immobilization was monitored for 7 h to determine the most appropriate immobilization time at pH 6 and 30 °C (Figure 1a,b). It was determined that purified PL enzyme was connected to magnetized lily flower with γ-Fe_3_O_4_ NPs support material after 60 min at the rate of 86.4% (Figure 1c). The pectin lyase showed maximum binding efficiency of 85% with calcium alginate beads in a previous study [13]. Similarly, the binding rate was found to be 87.2 in the study of Onem and Nadaroglu [12].

### 2.4. Biochemical Properties of Free and Immobilized Pectin Lyase

#### Effect of Temperature and pH

The optimum temperature for free and immobilized PL activity was determined by carrying out the standard assay after 60 min incubation in pH 6 at for temperatures ranging from 0 to 90 °C.

**Figure 2:**
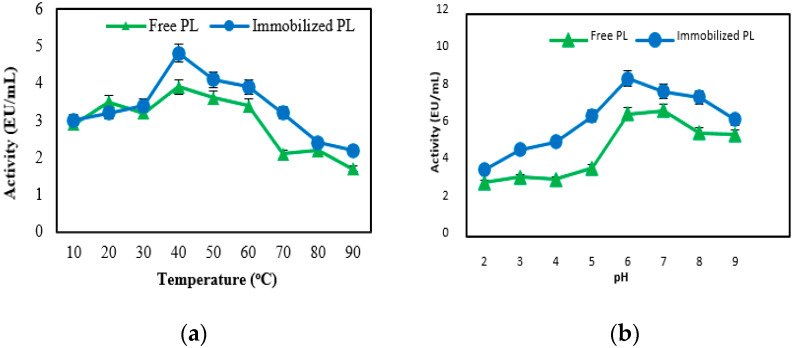
The effect of temperature (**a**) and pH (**b**) on activities of free and immobilized pectin lyase enzymes.

In each case, the substrate was pre-incubated at the desired temperature for 5 min. The maximum enzyme activity for free and immobilized pectin lyase was found at 40 °C. But, it was determined that both their activities decreased in higher temperatures (Figure 2a). The pH optimum of the free and immobilized PL was measured in pH changing from 2.0 to 9.0 at 30 °C for 90 min, using different buffers. The optimum pH for the free and immobilized pectin lyase enzyme was found to be 6.0 (Figure 2b). This value was obtained by taking into consideration the pH, temperature, and time in which the enzyme is stable (Figure 1).

There was no difference between free and immobilized enzymes due to changes in pH and temperature. It was determined that the optimum pH and optimum temperature values of pure pectin lyase obtained from different sources such as fungi and bacteria are generally between pH 4.5–10 and 30–70 °C [7,28,29,30,31]. It can be said that these values are consistent with our results.

### 2.5. pH Stability and Thermal Stability

Their natural origin and biocompatibility of the biomaterials, minimize negative effects on the structure and properties of enzymes, thus immobilized proteins retain their high catalytic activity against pH and temperature changes [16]. Enzyme stability, which is expected in the use of biomaterials as support materials, has been observed here also.

**Figure 3:**
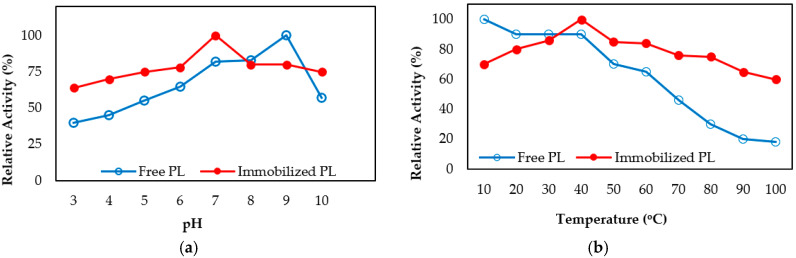
pH (**a**) and temperature (**b**) stability of free and immobilized pectin lyase on magnetized lily flower with γ-Fe_3_O_4_ NPs.

Free and immobilized pectin lyase enzymes were incubated at different pH ranges for 1 h at 4 °C. Acetate buffer for pH 3–5, phosphate buffer for 6–7, Tris/HCl buffer for 8–9 were used. Then the activities were measured. The results obtained are shown in (Figure 3a). The data show that free enzyme maintained 40% rate of its activity while immobilized enzyme was maintaining 64% rate of its activity at pH 3. Also, free pectin lyase maintained 57% rate of its activity while immobilized pectin lyase maintained 75% rate of its activity at pH 9.0. As seen from the results, immobilized pectin lyase enzyme activity was more stable against different pH values according to free enzyme (Figure 3a). The thermal inactivation rates of the soluble and immobilized enzyme were studied in the range of 10 to 100 °C at pH 6.0 in 50 mM Na-phosphate buffer. The thermostability of immobilized pectin lyase was measured by comparison to free pectin lyase as shown in (Figure 3b). The free enzyme lost about 80% of its activity at 90 °C after 1 h of heat treatment, while immobilized pectin lyase lost only 35% of its activity. It has been also observed that immobilized pectin lyase activity was preserved at high temperatures (Figure 3b).

pH stability and thermal stability of the free and immobilized pectin lyase enzymes have been preserved at high-low temperatures and pH values. It has been seen that immobilized PL enzyme generally increases thermal and pH stability compared to soluble enzymes as in previous studies [8,9,10,11,12,13,14,15,16].

### 2.6. SDS-PAGE Electrophoresis

SDS-polyacrylamide gel electrophoresis was performed to check the purity and subunit number of the pectin lyase enzyme purified by triple phase precipitation (TPP) from *Pseudomonas putida.*

**Figure 4:**
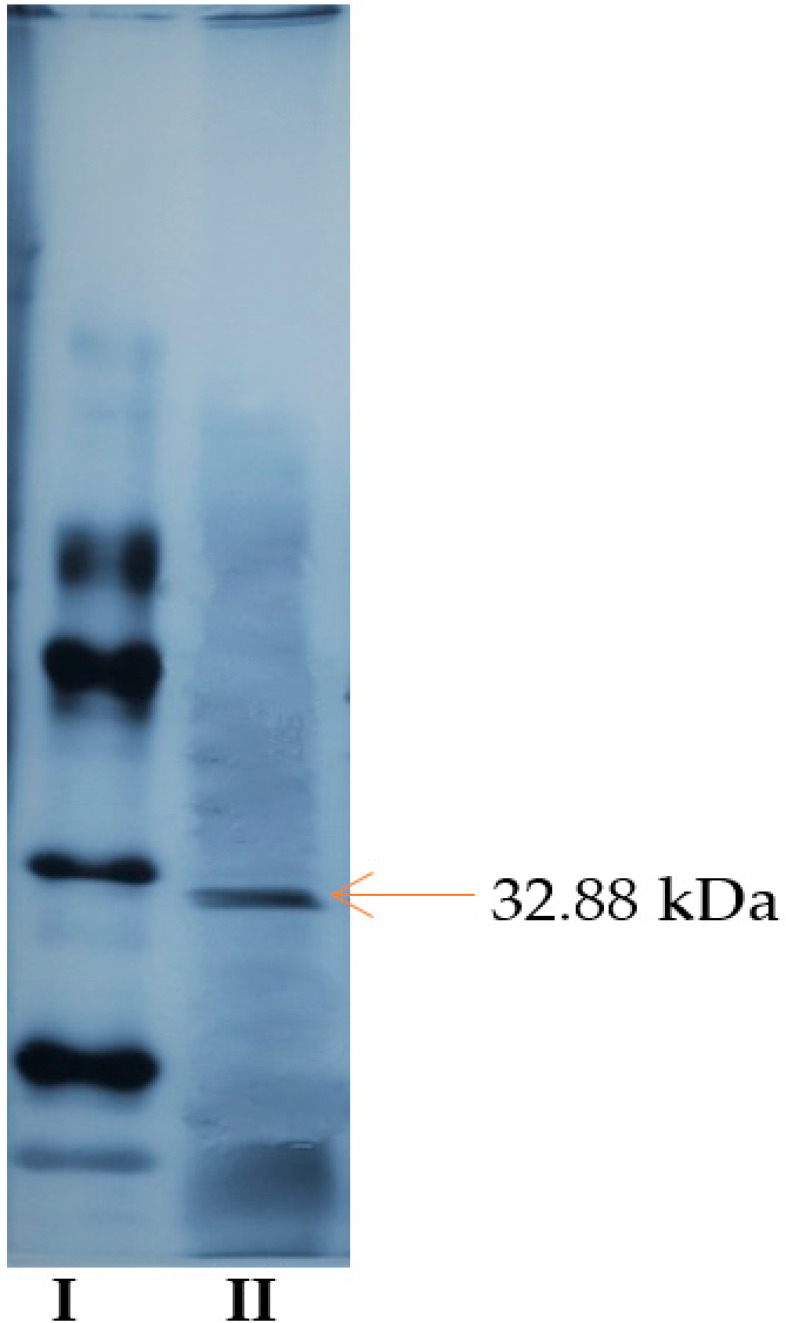
SDS-PAGE electrophoresis gel image of standard proteins (**I**) and purified pectin lyase enzyme from *Pseudomonas putida* (**II**).

As the gel images of PL enzyme obtained from *Pseudomonas putida* bacteria were compared with standard protein, single protein band was obtained and molecular weight was determined as 32.88 kDa (Figure 4). PL enzymes produced from *Geobacillus stearothermophilus* [1], *Bacillus pumilus* [2], *Aspergillus flavus* [27], and *Penicillum chrysogenum* [30] have 36 kDa, 25 kDa, 38 kDa, and 31 kDa molecular weight respectively.

### 2.7. Structural Characterization of Support

**Figure 5:**
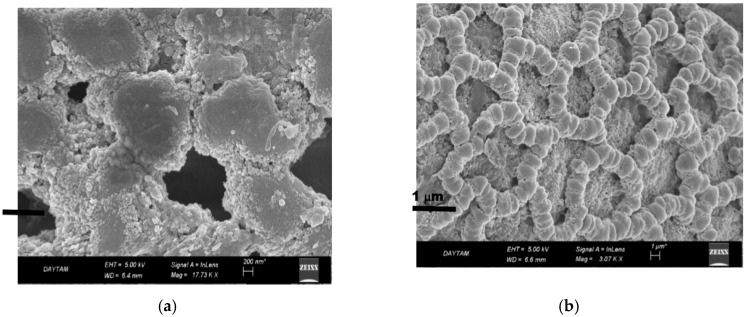
SEM images of immobilized PL on magnetite- Lily flower NPs (**a**,**b**).

The structure of immobilized PL on magnetite-lily flower NPs in SEM images are shown in Figure 5. The distribution of Fe_3_O_4_ NPs on the surface is clearly seen from SEM images. In addition, from the SEM image, it was observed that the support material’s structure was appropriate to modify nanomaterials and to immobilize PL enzyme. In addition, it had a large size that provides support and structural stability.

Intermediate reagents are often used to create strong cross-links in enzyme immobilization. The most commonly used bifunctional cross-linking reagent is glutaraldehyde because it is cheap and can easily bound to enzymes [16]. For this study, the lily flowers were activated with glutaraldehyde, thereby increasing the roughness of the surface after immobilization of the enzyme via the glutaraldehyde intermediate arm, allowing the enzyme to bind to the surface [24] (Figure 5).

### 2.8. X-ray Diffraction (XRD) Analysis

**Figure 6:**
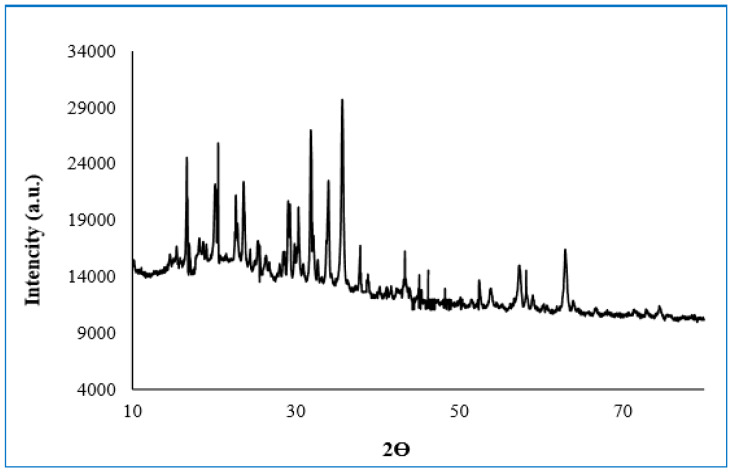
XRD patterns of IM-PL on magnetic lily NPs.

XRD patterns of free enzyme magnetite-lily flower NPs; purified pectin lyase immobilized magnetite- lily flower NPs are illustrated in Figure 6. The XRD pattern of lily flower exhibits broad diffraction peaks at 2θ = 16.65°, 20.51° 23.58°, 29.31°, 31.86°, 33.98°, 35.72°, and 63.08° which are typical fingerprints of Fe_3_O_4_ structure [31]. Energy distribution X-ray analysis spectroscopy (EDX) revealed the presence of elements of free and immobilized pectin lyase with magnetite-lily flowers NPs structures in Figure 6.

### 2.9. FT-IR Analysis

**Figure 7:**
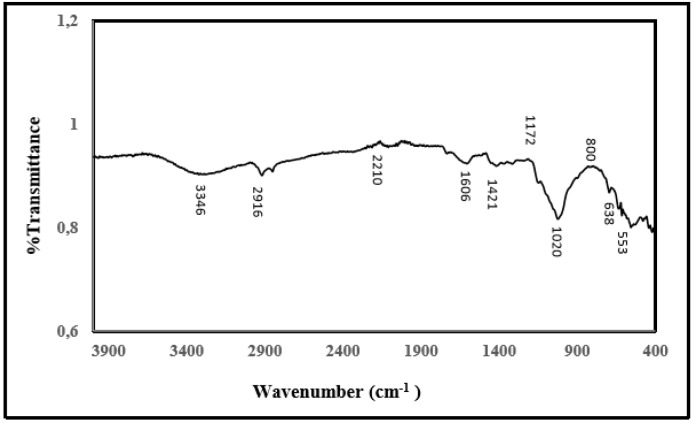
FT-IR spectrum of modified lily flower with nano magnetite and purified pectin lyase immobilized modified lily flower with nano magnetite.

Figure 7 shows the FT-IR spectrum of the structure of the IM-PL enzyme on the magnetic lily flower support material. It is seen from the spectrum that the characteristic absorption of Fe-O bond of Fe_3_O_4_ structure is 534 cm^−1^ and 638 cm^−1^, and the characteristic absorption of -OH bond is 3390 cm^−1^. The vibrations at 1421cm^−1^ and 1606 cm^−1^ are characteristic peaks of the COO-Fe bond which may be due to the reaction of hydroxide radical groups on the Fe_3_O_4_ surface with the carboxylate anion contained in the cellulosic structure of the lily flower. The peaks at 2850 cm^−1^ and 2916 cm^−1^ are due to vibration of the alkyl groups (at -CH_2_ and -CH_3_) in the plant’s long alkyl chain. Figure 7 shows the shift bond between OH and NH from 3419.79 to 3437.15 cm^−1^ for tensile vibration and from 1514.12 to 1560.41 cm^−1^ for NH_2_ bending.

The results of FT-IR spectrums indicated that lily flower was coated with the magnetic Fe_3_O_4_ nanoparticles and pectin lyase enzyme was immobilized onto the magnetic Fe_3_O_4_–lily flower nanoparticles successfully.

### 2.10. Application of Immobilised Pectin Lyase Enzyme in Juice Clarification

It was presented here how free and immobilized PL enzymes produced and purified from *Pseudomonas putida* affect the degradation rate and clarity of the juice production process used in fruit juice production. In fruit juice production process, grape (black), peach, apple (red), and plum (black) fruits obtained from local markets were used as puree.

Polysaccharides and colloids such as hemicellulose and pectin cause high viscosity in juices. Hydrolysis of these materials by enzymes allows this viscosity to be reduced [32]. It has been determined that the less viscous and clear juices can be formed by hydrolysis of the pectin by pectinases. While turbidity in fruit juices may be a desired feature in some cases (tomato juice, orange juice), it is sometimes preferred by the consumer to be clear and cloudless (such as sour cherry, pomegranate etc.), [33]. Viscosity reduction of fruit juice by enzymatic hydrolysis of pectin was illustrated by Urlaub (1996) [34]. For this purpose, % disintegration rate was calculated by comparison with control for amount of dry matter remaining after filtration of 10 g fruit. In addition, the effects of free and immobilized PL enzymes on grape (black), plum, peach and apple’s breakdown rates and the amount of juice obtained and clarification rates were determined. Increased volume values and absorbance changes in the filtrates were determined and the results are shown in Table 3.

The reduction of all fruit residues are observed, but especially a significant decrease was observed in the dry weight values of apple and black plum, and as a result, an increase in the juice volume was determined (Table 3). In the study of Dey and Banerjee (2014) [33], it was also determined that the amount of pectin in the fruit juice decreased after the juice was clarified with the enzyme. At the end of this study; immobilized PL was found to be more effective than free PL enzyme in clarifying some juices (Table 3).

**Figure 8:**
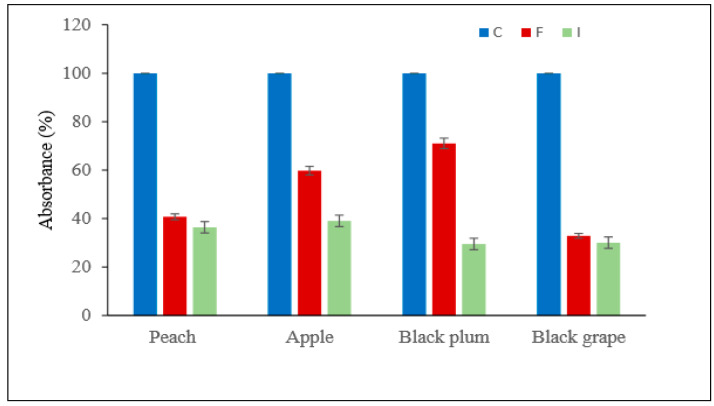
Absorbance% changes in all fruit juice filtrates control, treated with free and immobilized pectin lyase.

In addition, in this research, the absorbance changes in all filtrates were determined by measuring the optical densities at 660 nm and the results are shown in Figure 8 as reduction%. IMB-PL was found to be much more effective than did free PL enzymes in the clarification of different fruit juices (Figure 8). The magnetized IMB-PL reduced the degree of turbidity at a higher rate and caused an increase in the volume of different fruit juices such as results of the Demir et al. (Figure 8) [5]. Similarly, during clarification of apple juice, the viscosity reduction were reported by Xu et al. (2014), Yuan et al. (2011), Singh and Gupta (2004) and Busto et al. (2006) (respectively, %38.8, ~%4.5, ~%36 ve ~%35) [8,35,36,37].

## 3. Conclusions

As a result, pectin lyase purified from *Pseudomonas putida* bacterium was magnetized by using Fe_3_O_4_. Then, it was cross-linked with the self-grown lily flower that is easily accessible in nature via L-glutaraldehyde. So obtained magnetized IMB-PL was used in juice clarification. The nontoxic of the lily flower, which is the support material, may make it easier to use in food applications. In this study, for the first time, it was used as a flower support material for immobilization of the pectin lyase enzyme, which was highly purified by triple phase precipitation technique. The fact that magnetic immobilized PL with a high degree of immobilization such as the rates specified in the literature is effective in juice clarification can be considered as a striking result. It may be thought that these result are based on the larger surface area of the nanomagnetite immobilized pectin lyase enzyme. As a result, it can be stated that the nanomagnetite immobilized PL structure can be easily used in different industrial fields, especially in the food industry in a stable structure without inhibiting it. The immobilized enzyme covalently bound to NPs bound to Fe_3_O_4_ modified lily flowers is more resistant to temperature than the free enzyme. It can be concluded that the covalent binding of the enzyme to magnetite-lily flower NPs causes conformational changes in the pectin lyase enzyme and therefore may be more resistant to some stress factors. This shows that immobilized enzyme is more advantageous for use in applications than free enzyme and exhibited good reusability for pectin hydrolysis.

## 4. Materials and Methods

### 4.1. Bacterial Isolation

In this study, microorganism was isolated from parsley obtained from Erzurum local markets. A sample was prepared from the parsley sample at 10 µg/mL concentration under aseptic condition. Subsequently, serial dilutions from 10 to 10^−8^ were prepared using physiological serum. Foods from each dilution were spread onto agar medium and then incubated at 34 °C for 48 h. After incubation, nutrient agar medium was drawn from typical colonies developed and drawing was made by drawing 3 phases. This was continued until pure colonies were obtained. The purified colony was then transferred to nutrient broth containing 20% glycerol solution. It was then stored as a stock culture at −80 °C.

### 4.2. Identification Tests

In this study, among general identification tests, Gram staining and catalase tests were performed on isolated microorganism. According to the results of microbiological analysis; it was found to be Gram positive and catalase positive.

### 4.3. Identification of Microorganism

After isolation of microorganisms from parsley has been carried out, obtained pure cultures were identified at species level. For this purpose, after DNA isolation from pure culture was done, identification was made by means of the sequence analysis of intergenic spacer regions (ISR) of genes 16S rRNA and 16S-23S rRNA, which is considered as the most reliable method in microbial identification.

### 4.4. Genotype Characterization of Bacteria

For the sequence analysis of the 16 S rRNA genes, universal primers LPW57 (5 W-AGTTTGATCCTGGCTCAG-3′) and LPW205 (5′-CTTGTTACGACTTCACCC-3 T) [38] were used. In the amplification of the ISR region, 16-1A (GTCGGAATCGCTAGTAATCG) and 23-1B (GGGTTCCCCCATTCGGA) [39] universal primers were used. Sequence analysis was made by Medsantek company (Istanbul, Turkey). The results of complete sequence analysis of 16S rRNA and 16S-23S rRNA intergenic spacer region (ISR) were compared with those of other bacterial sequences in GenBank (http://blast.ncbi.nlm.nih.gov/blast.cgi) and similarity ratio was determined [40]. According to this ratio, the bacterium was compared with the other bacterial series in GenBank, and this isolate was found to be similar with *Pseudomonas putida* bacteria by 99%.

### 4.5. PL Enzyme Production with Solid Culture Fermentation

PL enzyme from *Pseudomonas putida* bacteria produced in solid culture medium was purified and characterized by three-phase precipitation (TPP) technique [11]. For this purpose, n-butanol optimization was carried out, followed by ammonium sulfate optimization. Briefly, the amount of ammonium sulphate in the reaction medium was kept constant and different n-butanol (1:0.5, 1.0:1.0, 1.0:1.5, 1.0:2.0) precipitation was performed in three phases. Next, the precipitate middle phase was dissolved in 1 mL of 0.05 M phosphate buffer (pH 8.0) and dialyzed against the same buffer for 3 h.

### 4.6. Purification of PL from Pseudomonas Putida Using TPP Method

PL enzyme, which was produced extracellularly from *Pseudomonas putida* bacterium, was purified by triple phase precipitation (TPP) method [11,12]. For the optimization of ammonium sulfate, PL was precipitated by adding different ammonium sulfate (20%, 40%, 60%, and 80% ammonium sulfate) to the homogenate medium at the optimum n-butanol ratio (1.0:0.5%). Then, the percentages were determined according to the optimum amount of n-butanol and ammonium sulphate. Purification of PL enzyme was carried out according to the parameters obtained. PL enzyme activity was determined by color derivation [41].

### 4.7. Pectin Lyase Activity Assay

Pectin lyase activity was determined by color derivative formation method [41]. The determination of the PL activity was carried out by monitoring the increase in absorbance at 550 nm using BioTek’s EpochTM Multi-Volume Spectrophotometer, (BioTek Instruments, Winooski, VT, USA). The blank sample was prepared using phosphate buffer instead of enzymes. The one unit of activity (1 EU) was defined as the amount of enzyme converting 1 μmol substrate into product in 1 min at 25 °C under standard conditions. All experiments were repeated three times.

### 4.8. Preparation of Nano-Magnetic Lily Flowers (N-MLf) and Immobilization of Purified Pectin Lyase

**Figure 9:**
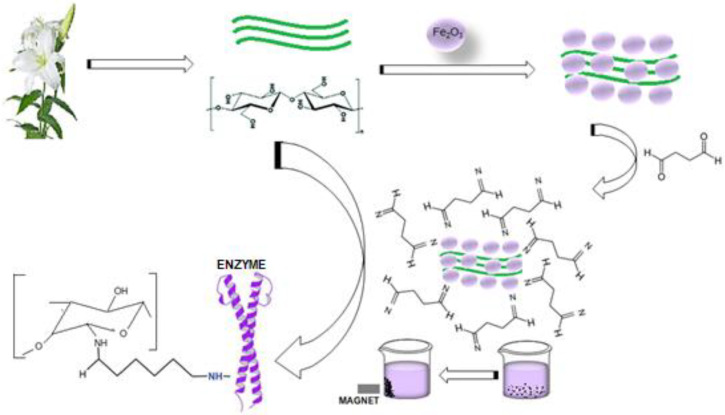
Reaction mechanisms of the cellulosic structures of LFs (*Lilium candidum* L.) modified with glutaraldehyde and immobilization of pectin lyase enzyme to support material.

The enzyme was covalently immobilized to the cellulosic structures of LF (*Lilium candidum* L.) via the L-glutaraldehyde spacer (Figure 9). In the immobilization process, first of all, the lily flowers were disintegrated with a blender in distilled water and the liquid was removed by filtration. Then the solid material was stirred with 1 N NaOH for 1 h and washed with distilled water until neutralization. The magnetic properties of the cellulosic surface of the lily flowers were determined by mixing with Fe_3_O_4_ dispersed in distilled water overnight. Finally, it was washed with distilled water to remove non-binding nanoparticles, and the wet precipitate was dried at 40 °C for 72 h. Thus LF was made magnetic by being modified with Fe_3_O_4_ nanoparticles. The magnetic lily flower (*N*-MLf) was stored at 4 °C for use in experimental studies. Purified pectin lyase enzyme purified from *Pseudomonas putida* was immobilized to the surface of the *N*-MLf activated with %1 L-glutaraldehyde at appropriate incubation medium (30 °C for 60 min). Then, they were washed with distilled water under vacuum to remove the non-binding glutaraldehyde from the medium, respectively (Figure 9). The amount of protein was determined in the reaction medium at different time intervals, by using Warburg and Bradford methods and the amount of binding pectin lyase enzyme was determined against time [42].

### 4.9. Protein Determination

Protein concentration of samples was determined spectrophotometrically by using the Bradford method [42]. Bovine serum albumin (BSA) was used as a standard.

### 4.10. Preparation of Nanomagnetite Flowers of Lily (Lilium candidum L.)

The magnetic property of surface of the cellulosic structures of LFs (*Lilium candidum* L.) was provided by treating with dispersed γ-Fe_3_O_4_ in distilled water. 0.10 g γ-Fe_3_O_4_ nanoparticles were fully dispersed in the lily flowers solution under ultrasonic vibration for 30 min and then it was washed with distilled water to remove the non-binding nanoparticles. Thus, immobilized PL (IMB-PL) was made magnetic by being modified with γ-Fe_3_O_4_ nanoparticles [43].

### 4.11. Determination of Optimum Conditions for Immobilization of Purified PL Enzyme onto Modified Lily Flowers with γ-Fe_3_O_4_ NPs

In order to find the highest rate of immobilization occurred in which pH, immobilization was made in the range of pH from 2.0 to 8.0. Also, in order to find the highest rate of immobilization occurred in which temperature, immobilization was carried out at 10, 15, 30, 40, 50, 60, 70, and 80 °C in previously determined pH.

The pectin lyase binding efficiency (E) is defined as follows:

E = (C_1_ − C_0_)/C_1_ (1)

C_1_ and C_0_ are the amounts of pectin lyase protein in the solution before and after immobilized, respectively.

The activity amount of the immobilized pectin lyase was calculated as follows:

Immobilized pectin lyase = (A_1_ − A_0_)/A_1_ (2)

A is the activity of the immobilized pectin lyase and A_1_ and A_0_ are the activities of the free pectin lyase in solution before and after immobilization, respectively.

### 4.12. Determination of the Optimum pH and Stable pH

The effect of pH on the activity and stability of free pectin lyase and immobilized pectin lyase was identified. Therefore, when using pectin as substrate, sodium acetate buffer (20 mM) pH range 3.0 to 5.0, phosphate buffer (20 mM) pH range 6.0 to 7.0, Tris-HCl buffer (20 mM) At pH 8 and in the pH range between 9.0 and 10.0, phosphate buffer (20 mM) was applied to determine the effect of pH on free pectin lyase and immobilized pectin lyase activity. The pH stability was tested by activity monitoring by incubating at 4 °C in the above-mentioned buffer solutions at different pHs at a concentration of 20 mM for 1 week.

### 4.13. Determination of Optimum and Stable Temperature Values

PL activity measurements were performed at 10–90 °C to determine the temperature at which the enzyme showed optimal activity. Water bath was used for activity measurements at different temperatures. The optimum temperature of the enzymes was determined by the results obtained. To determine the stable temperature of the enzyme. Efficacy measurements were made at a temperature range of 10–90 °C. Separate sample and blind reactions were generated for each temperature test, and efficacy measurements were performed every 15 min for 2 h. All measurements were performed against the blind sample [2].

### 4.14. Characterization of Free and Immobilized Enzymes

Vmax and K_M_ were determined for the pectin substrate of both free and immobilized pectin lyase enzymes. By using different substrates (pectin, locust bean gum, and chitin), PL activity measurements in the concentrations range of 0.25 to 1.5 mM were made and Lineveawer burk plots were plotted. Then, maximum reaction rate (Vmax) and Michaelis-Menten constant (K_M_) values were determined.

### 4.15. Application of Immobilized Enzyme in Fruit Juice Clarity

The effects of free and immobilized pectin lyase enzymes on the clarity and disintegration of grape (black), apple (red), and plum (black) juices were investigated. The fruits were homogenized with a blender. The purees were then diluted 1/1 with distilled water in the beakers and 2 mL of enzyme and homogenate were added to each group separately. Control, free and immobilized enzyme addition groups were formed. Experiments using free enzyme and immobilized enzyme were compared with control experiment in juice extraction processes. For control groups, distilled water was used instead of enzymes. The sample prepared by adding 2 mL of pure water to 10 g of fruit puree was used as standard. The beakers were incubated for 5 h with stirring in a 50 °C water bath. After cooling the purees, the juices were obtained by vacuum filtration through filter paper. After the juice was removed, the remaining fruit extract was dried at 105 °C until constant weighing [44].

Experiments using free enzyme and immobilized enzyme in fruit juice extraction processes were compared with the control experiment that used pure water instead of enzymes.

### 4.16. Structural Characterization of Support Material

Structural and morphological properties of free and immobilized pectin lyase magnetized by modifying with Fe_3_O_4_ NPs were investigated using scanning electron microscopy (SEM) (JSM 6400; JEOL Ltd., Tokyo, Japan), X-ray diffraction (XRD) analysis (Rigaku- Miniflex X-Ray Diffraction System; Rigaku, Neu-Isenburg, Germany), and Fourier transform infrared spectroscopy (FTIR) (VERTEX 70v; Bruker, Billerica, MA, USA; and Mattson 1000 FTIR spectrophotometer; Midland, ON, Canada) techniques.

### 4.17. Statistical Analysis

Data are presented as the mean ± standard deviation of each treatment. Data were analyzed for statistical significance using analysis of variance (ANOVA) followed by the Tukey test (p > 0.05) (IBM SPSS Statistics v20, Chicago, IL, USA).

## Figures and Tables

**Table 1:** The purification process of purified pectin lyase enzyme from *Pseudomonas putida.*

Enzyme Fraction	Volume (mL)	Activity (EU/mL)	Total Activity (EU/mL) %	Specific Activity (EU/mL)	Protein (mg protein/mL)	Purification Fold
**Crude extract**	60	263.2 ± 0.01	15792 100	0.17	1530.9 ± 0.011	-----
**n-Butanol (1:0.5)**	20	224.6 ± 0.04	4492 85	1.16	192.4 ± 0.0171	6.82
**1. (NH4)_2_SO_4_ (%60)**	20	208.5 ± 0.011	4170.8 79.2	5.49	37.97 ± 0.016	32.3
**2. (NH4)_2_SO_4_ (%65)**	20	191.4 ± 0.012	3710 73	37.52	5.1 ± 0.014	220.7

**Table 2:** Vmax and KM values of pure PL and (Nano Magnetit Flower- Pectin lyase) NMF-PL enzymes for the substrates of pectin, locust bean gum, and chitin.

	Pectin		Locust Bean Gum		Chitin	
	V_max_	K_M_	V_max_	K_M_	V_max_	K_M_
	(µmol/Lmin)	(mg/mL)	(µmol/Lmin)	(mg/mL)	(µmol/Lmin)	(mg/mL)
**Pure PL**	18.62	1	1.45	0.65	1.26	0.5
**NMF-PL**	23.20	0.86	1.98	0.61	1.34	0.42

**Table 3:** Degradation rates of dry matter in fruits control, treated with free and immobilized pectin lyase (PL) enzyme. DW: dry weight %D: decrease rate.

Fruit (10 g)	Dry Weight			Increasing Volume (mL)		
	Control *	Free PL	Immobilized PL	Control	Free PL	Immobilized PL
	DW(g) %D	DW(g) %D	DW(g) %D			
**Peach**	0.244 2.4	0.168 1.7	0.202 0.2	7.0	7.0	8.5
**Apple**	0.161 1.6	0.152 1.5	0.144 1.4	8.0	8.5	9.2
**Black Plum**	0.233 2.3	0.19 1.9	0.102 1	7.5	7.0	9.2
**Black Grape**	0.121 1.2	0.114 1.1	0.065 0.6	7.0	7.0	8.0

* Control; pure water was used instead of enzymes.

## References

[1] Demir N., Nadaroğlu H., Tasgın E., Adıgüzel A., Gulluce M. (2011). Purification and Characterization of a pectin lyase produced by *Geobacillus stearothermophilus* (Ah22) and fruit juice application. Ann. Microbiol..

[2] Nadaroglu H., Taskin E., Adiguzel A., Gulluce M., Demir N. (2010). Production of a novel pectin lyase from *Bacillus pumilus* (P9), purification and characterisation and fruit juice application. Rom. Biotechnol. Lett..

[3] Márquez A.L., Zavala-Páramo M.G., López-Romero E., Calderón-Cortés N., López-Gómez R., Conejo-Saucedo U., Cano-Camacho H. (2011). Cloning and characterization of a pectin lyase gene from *Colletotrichum lindemuthianum* and comparative phylogenetic/structural analyses with genes from phytopathogenic and saprophytic/opportunistic microorganisms. BMC Microbiol..

[4] Fontana R.C., Silveira M.M. (2012). Influence of pectin, glucose, and pH on the production of endo- and exo-polygalacturonase by *Aspergillus oryzae* in liquid medium. Braz. J. Chem. Eng..

[5] Demir N., Nadaroglu H., Demir Y., Isık C., Taskin E., Adiguzel A., Gulluce M. (2014). Purification and characterisation of an alkaline pectin lyase produced by a newly isolated *Brevibacillus borstelensis* (P35), and its applications in fruit juice and oil extraction. Eur. Food Res. Technol..

[6] Mohamadi A.S., Shahbazi S., Behgar M., Fard S.M., Askari H. (2014). A study of pectinase enzyme activity changes in gamma- irradiated *Trichoderma reesei* mutants. Int. J. Farm. Allied Sci..

[7] Pedrolli D.B., Monteiro A.C., Gomes E., Carmona E.C. (2009). Pectin and Pectinases: Production, Characterization and Industrial Application of Microbial Pectinolytic Enzymes. Open Biotechnol. J..

[8] Busto M.D., García-Tramontín K.E., Ortega N., Perez-Mateos M. (2006). Preparation and properties of an immobilized pectinlyase for the treatment of fruit juices. Bioresour. Technol..

[9] Sorrivas V., Genovese D.B., Lozano J.E. (2006). Effect of pectinolytic and amylolytic enzymes on apple juice turbidity. J. Food Proc. Preserv..

[10] Magro L.D., Silva de Moura S., Backes B.E., Weber de Menezes E., Valmir Benvenutti E., Nicolodi S., Kleind M.P., Fernandez-Lafuentee R., Rodriguesa R.C. (2019). Immobilization of pectinase on chitosan-magnetic particles: Influence of particle preparation protocol on enzyme properties for fruit juice clarification. Biotechnol. Rep..

[11] Sharma A.K., Gupta M.N. (2002). Three phase partitioning of carbohydrate polymers: Separation and purification of alginates. Carbohydr. Polym..

[12] Onem H., Nadaroglu H. (2018). Immobilization of Purified Phytase Enzyme from Tirmit (*Lactarius volemus)* on Coated Chitosan with Iron Nanoparticles and Investigation of Its Usability in Cereal Industry. Iran. J. Sci. Technol. Trans. Sci..

[13] Kohli P., Gupta R. (2019). Application of calcium alginate immobilized and crude pectin lyase from *Bacillus cereus* in degumming of plant fibres. Biocatal. Biotransform..

[14] Sheldon R.A. (2007). Enzyme immobilization: The quest for optimum performance. Adv. Synth. Catal..

[15] Mateo C., Palomo J.M., Fernandez-Lorente G., Guisan J.M., Fernandez-Lafuente R. (2007). Improvement of enzyme activity, stability and selectivity via immobilization techniques. Enzyme Microb. Technol..

[16] Zdarta J., Meyer A.S., Jesionowski T.I.D., Pinelo M. (2018). A General Overview of Support Materials for Enzyme Immobilization: Characteristics, Properties, Practical Utility. Catalysts.

[17] Ramirez H.L., Brizuela L.G., Iranzo J.U., Arevalo-Villena M., Perez A.I.B. (2016). Pectinase immobilization on a chitosan-coated chitin support. J. Food Process. Eng..

[18] Irshad M., Murtza A., Zafar M., Bihatti K.H., Rehman A., Anwar Z. (2017). Chitosan-immobilized pectinolytics with novel catalytic features and fruit juice clarification potentialities. Int. J. Biol. Macromol..

[19] Rehman H.U., Aman A., Silipo A., Qader S.A.U., Molinaro A., Ansari A. (2013). Degradation of complex carbohydrate: Immobilization of pectinase from *Bacillus licheniformis* KIBGE-IB21 using calcium alginate as a support. Food Chem..

[20] Horchani H., Aissa I., Ouertani S., Zarai Z., Gargouri Y., Sayari A. (2012). Staphylococcal lipases: Biotechnological applications. J. Mol. Catal. B Enzym..

[21] Vijayaraghavan K., Yamini D., Ambika V., Sravya Sowdamini N. (2009). Trends in inulinase production—A review. Crit. Rev. Biotechnol..

[22] Homaei A.A., Sariri R., Vianello F., Stevanato R. (2013). Enzyme immobilization: An update. J. Chem. Biol..

[23] Krajewska B. (2004). Application of chitin- and chitosan-based materials for enzyme immobilizations: A review. Enzyme Microb. Technol..

[24] Kurita K. (2001). Controlled functionalization of the polysaccharide chitin. Prog. Polym. Sci..

[25] Peter M. (1995). Applications and environmental aspects of chitin and chitosan. J. Macromol. Sci..

[26] Mehmood T., Saman T., Irfan M., Anwar F., Ikram M.S., Tabassam Q. (2018). Pectinase Production from *Schizophyllum commune* Through Central, Composite Design Using Citrus Waste and Its Immobilization for Industrial Exploitation. Waste Biomass Valorization.

[27] Yadav S., Yadav P.K., Yadav D., Yadav K.D.S. (2008). Purification and characterization of an alkaline pectin lyase from *Aspergillus flavus*. Process Biochem..

[28] Babagil A. (2018). Purification and characterization of pectin lyase enzymes from microorganism, preparation of enzymic Ca^2+^-hybrid nanoflower structure and the investigation of use in fruit juice clarification. Ph.D. Thesis.

[29] Lei Z., Bi S. (2007). The silica-coated chitosan particle from a layer-by-layer approach for pectinase immobilization. Enzym. Microb. Technol..

[30] Banu R., Kalpana A., Devi A.M., Gnanaprabhal G.R., Pradeep B.V., Palaniswamy M. (2010). Production and characterization of pectinase enzyme from *Penicillium chrysogenum*. Indian J. Sci. Technol..

[31] Ogawa K., Hirano S., Miyanishi T., Yui T., Watanabe T. (1984). A new polymorph of chitosan. Macromolecules.

[32] Cerreti M., Markosova K., Esti M., Rosenberg M., Rebros M. (2017). Immobilisation of pectinases into PVA gel for fruit juice application. Int. J. Food Sci. Technol..

[33] Dey T.B., Banerjee R. (2014). Application of decolourized and partially purified polygalacturonase and α-amylase. Braz. J. Microbiol..

[34] Urlaub R. (1996). Advantages of enzymatic apple mash treatment and pomace liquefaction. Fruit Process..

[35] Yuan P., Meng K., Huang H., Shi P., Luo H., Yang P., Yao B. (2011). A novel acidic and low-temperature-active endo-polygalacturonase from Penicillium sp. CGMCC 1669 with potential for application in apple juice clarification. Food Chem..

[36] Singh S., Gupta R. (2004). Apple juice clarification using fungal pectinolytic enzyme and gelatine. Indian J. Biotechnol..

[37] Xu S.X., Qin X., Liu B., Zhang D.Q., Zhang W., Wu K., Zhang Y.H. (2014). An acidic pectin lyase from *Aspergillus niger* with favourable efficiency in fruit juice clarification. Lett. Appl. Microbiol..

[38] Woo P.C.Y., Fung A.M.Y., Lau S.K.P., Yuen K.Y. (2002). Identification by 16S rRNA gene sequencing of *Lactobacillus salivarius* bacteremic cholecystitis. J. Clin. Microbiol..

[39] Forsman P., Tilsala-Timisjarvi A., Alatossava T. (1997). Identification of staphylococcal and streptococcal causes of bovine mastisis using 16S-23S rRNA spacer regions. Microbiology.

[40] Garbers I., Britz T.J., Witthuhn R.C. (2004). Ctypification and identification of the microbial consortium present in Kefir grains. World J. Microbiol. Biotech..

[41] Nedjma M., Hoffmann N., Belarbi A. (2001). Selective and sensitive detection of pectin lyase activity using a colorimetric test: Application to the screening of microorganisms possessing pectin lyase activity. Anal. Biochem..

[42] Bradford M.M. (1976). A rapid and sensitive method for the quantitation of microgram quantities of protein utilizing the principle of protein-dye binding. Anal. Biochem..

[43] Nadaroğlu H., Alayl Gungor A., Ince S. (2017). Synthesis of nanoparticles by green synthesis method. Int. J. Innov. Res. Rev..

[44] Soares M.M.C.N., Sılva R., Gomes E. (1999). Screening of bacterial strains for pectinolytic activity: Characterization of the polygalacturonase produced by *Bacillus sp.*. Rev. Microbiol..

